# Systems-Level Interventions to Disrupt Structural Racism and Improve Black Adolescent Health Outcomes: A Scoping Review

**DOI:** 10.3390/soc16040112

**Published:** 2026-03-27

**Authors:** Tamara Taggart, Simone Sawyer, Connor Mitchell, Marcy S. Ekanayake-Weber, Robert W. Faris, Nisha O’Shea, Luz E. Robinson, Belinda Woodard, Wan-Chen Lin, Yinuo Xu, Yutong Gao, Kate Nyhan, Dorothy L. Espelage

**Affiliations:** 1 Department of Urban-Global Public Health, Rutgers University, Newark, NJ 07102, USA; 2 Department of Social and Behavioral Sciences, Yale University, New Haven, CT 06510, USA; 3 Department of Sociology, University of California at Davis, Davis, CA 95616, USA; 4 Department of Anthropology, Center for the Elimination of Health Disparities, University at Albany State University of New York, Albany, NY 12222, USA; 5 Gillings School of Global Public Health, University of North Carolina at Chapel Hill, Chapel Hill, NC 27599, USA; 6 McCausland College of Arts and Sciences, University of South Carolina, Columbia, SC 29208, USA; 7 School of Education, University of North Carolina at Chapel Hill, Chapel Hill, NC 27599, USA; 8 Cushing/Whitney Medical Library, Yale School of Public Health, New Haven, CT 06520, USA

**Keywords:** Black adolescents, community, policy, interventions, structural racism, health

## Abstract

Structural racism and discrimination (SRD) is a fundamental cause of health inequities that emerge during adolescence and persist throughout adulthood. This scoping review systematically synthesizes the evidence on policy and community-level interventions designed to disrupt SRD exposure among Black adolescents and mitigate its impact on their health behaviors and outcomes. Following PRISMA-ScR guidelines, we searched five databases for peer-reviewed intervention studies published through October 2025. Of 3417 abstracts screened, 9 studies met inclusion criteria. We examined the study characteristics, theoretical frameworks, implementation strategies, and effectiveness of interventions targeting three primary mechanisms of SRD exposure for adolescents. The majority focused on neighborhood and social integration interventions, with limited representation of resource-based and school-based approaches. Culturally grounded, community-engaged interventions buffered SRD’s negative effects on mental health, empowered youth as change agents, and removed structural barriers to health-promotive resources. The review identified several gaps in the research, including methodological and theoretical rigor, geographic contexts, and follow-up. Findings underscore the potential of culturally grounded, multilevel interventions to reduce inequities across mental health, physical health, and social outcomes for Black youth. This review highlights the need to expand systems-level interventions that address the root causes of the persistent racial health inequities experienced by Black youth.

## 1. Introduction

Structural racism and discrimination (SRD) is a fundamental determinant of health. SRD is a driver of health inequities with historical roots extending from the colonial era through contemporary institutional policies, practices, and social norms [1,2]. In the United States (US), SRD manifests through the unequal distribution of resources, power, and opportunities within reinforcing inequitable social institutions to disadvantage Black Americans while advantaging White Americans [3]. These inequities are evident in early childhood, but expand dramatically during adolescence (ages 10–19 years), a critical developmental period that can significantly impact health behaviors and outcomes that persist across the life course, despite considerable advances in health promotion and medical innovation [4–7].

Since the civil rights era, public health interventions and policies have been developed with the goal of reversing the devastating effects of SRD in the US [3]. Yet, most of these interventions focus on individual and interpersonal mechanisms that, although important, place the burden of change on those experiencing SRD [3]. While such approaches are needed, they have not significantly advanced population health equity. There remains a critical need for systems-level interventions that reduce and ultimately eliminate SRD and its impact on adolescent health [6]. Despite growing public health recognition of this need, the evidence base for policies and community-level interventions that disrupt, rather than mitigate, SRD remains underdeveloped [3,8]. Below, we describe three primary intervention mechanisms that show promise for disrupting SRD’s impact on Black adolescent health: (1) resource-based policies and interventions; (2) neighborhood and social integration interventions; and (3) school-based interventions.

Resource-based policies and interventions address the resource imbalances and socioeconomic inequities perpetuated by SRD [9,10]. For example, guaranteed basic income and cash transfer programs reduce psychosocial distress associated with economic and housing instability among Black adolescents, thereby reducing the risk of poor mental health and substance use [11,12]. Similarly, interventions that increase access to and the affordability of higher education and vocational training (e.g., tuition assistance programs, community college promise initiatives, and other programs that provide tuition-free access to higher education for low-income and under-resourced community members) improve long-term economic stability and well-being [13–15]. Housing mobility programs can also reduce exposure to disadvantaged neighborhoods and associated health risks, including substance use, sexual risk behaviors, violence, and mental health problems [16–18]. While these resource-based approaches show promise, evidence of their effectiveness in disrupting SRD and its health effects for Black adolescents has not been systematically synthesized.

Neighborhood and social integration interventions operate through a different mechanism to disrupt SRD. Neighborhoods play a pivotal role in adolescent exposure to SRD, and thus, interventions targeting structural characteristics and social processes within neighborhoods influence adolescent health and development. Access to quality housing, healthy food, and safe community spaces, along with neighborhood social cohesion and collective efficacy, are associated with positive community health and well-being [19]. Black adolescents in economically disadvantaged neighborhoods characterized by violence and limited social resources have fewer social ties and trusted adults in their lives, and feel less connected to their communities, which is negatively associated with health [20–22]. However, high-quality, comprehensive, developmentally and culturally relevant resources in neighborhoods can serve as protective factors for Black adolescents in under-resourced environments [23,24]. These resources are often provided through community-based organizations (CBOs) that develop spaces for adolescents to feel a sense of belonging, facilitate intergenerational relationships, and offer programming that enhances their critical consciousness and civic engagement [23,24]. Evidence suggests these approaches improve health outcomes, yet questions remain on how to scale CBO resources across diverse neighborhood contexts and measure their specific effectiveness in disrupting exposure to SRD.

Schools represent the third intervention mechanism. School discipline policies are a primary mechanism through which SRD operates [25] with prior research showing that Black students face disproportionate exposure to exclusionary practices, including reduced placement in advanced tracks, higher transfer rates to alternative schools, and increased law enforcement referrals [26–28]. These disparities lead to poor mental health, worse academic outcomes, increased substance use, and other risk behaviors that persist through adulthood, with lasting effects on economic stability and social mobility [29–31] Although alternative approaches such as restorative justice practices, trauma-informed care, and enhanced behavioral health support have been implemented in some school settings, there remains a limited understanding of how these policies and community-level interventions should be designed and implemented to effectively disrupt SRD in schools.

In this scoping review, we systematically synthesize the evidence on policy and community-level interventions designed to disrupt SRD exposure among Black adolescents and mitigate its impact on their health behaviors and outcomes. Unlike prior reviews that focus on individual-level interventions, we examine systems-level approaches aimed at addressing the structural drivers of health inequities [32,33]. This scoping review is guided by these central questions:

1. What types of policy and community-level interventions have been developed to address SRD exposure among Black adolescents living in the US, and what theoretical frameworks underpin these interventions?
2. What are the intervention components, implementation strategies, and reported effectiveness of these interventions?
3. What gaps exist in the current evidence base for systems-level approaches, and what opportunities remain for innovation and improved implementation?

Our synthesis focuses on the three intervention mechanisms described previously: (1) resource-based interventions that address socioeconomic inequalities (e.g., cash transfers, housing vouchers, improved public transportation, educational opportunities); (2) neighborhood and social integration interventions that expand resources and diversify social networks (e.g., community-based organization programs, social programs, public space development); and (3) school-based interventions that reduce exclusionary discipline practices and provide health-promotive resources in school settings (e.g., restorative justice approaches, trauma-informed practices, enhanced mental health support in schools).

## 2. Methods

### 2.1. Search Strategy

This scoping review followed Arksey & O’Malley’s five stages of a scoping review and aligned with PRISMA-ScR guidelines [34,35]. This review was designed by domain experts (TT and SS) in consultation with a health sciences librarian (KN), who provided guidance for developing the search strategy, search terms, and identifying academic databases. We searched PubMed, PsycINFO (Ovid), Scopus, ERIC (Ovid), and EconLit (ProQuest) for research published in English (in print or electronically) through 23 October 2025. The search utilized keyword searches of the title and abstract fields, as well as controlled vocabulary, to identify community and policy interventions addressing SRD and its effects on adolescent health outcomes in research articles (see Supplementary Material: 2025-10-23 PubMed search history for Covidence and translations, for a full list of search terms and the search history). Search terms were informed by search terms used in other systematic reviews and in consultation with the domain experts and librarian [36,37]. Reference sections of relevant review articles were searched for any intervention studies not identified through the above search, but that potentially met the inclusion criteria for this review. We also conducted forward citation chaining of all included articles using Citation Chaser software [38] which draws on the citation network of The Lens.

### 2.2. Selection Criteria

Studies were included in the study if they were: (1) Intervention studies that documented the implementation and/or impact of policy and community level interventions that address or may affect SRD and measure effects on a health outcome; (2) Conducted in the United States; (3) Primarily focused on policy and community level intervention approaches including resource-based interventions, neighborhood and social integration interventions, or school-based interventions; (4) Measured outcomes relevant to adolescents (ages 10–19 years), regardless of whether adolescents are the direct intervention recipients, with a focus on Black populations (i.e., African American, Caribbean Black, African) where at least 51% of the sample is Black. The 51% threshold was selected to ensure selected interventions were relevant to Black people and findings could be applied to Black adolescents. The final inclusion criteria were that all articles be (5) Published in a peer-reviewed journal. We excluded: (1) Studies that do not evaluate, describe, or examine the implementation of an intervention; (2) Studies conducted outside of the U.S.; (3) Studies not written in English; (4) Commentaries, letters to the editor, opinion pieces, dissertations, study protocols, other systematic or scoping reviews, and feature articles (i.e., narrative-style journalistic pieces); (5) Studies focused exclusively on individual or interpersonal level interventions without policy or community-level components. Inclusion and exclusion criteria are outlined in Table 1.

### 2.3. Data Management and Extraction

Covidence, a systematic review data management program [39] was used for deduplication and to conduct title/abstract and full-text review. Our interdisciplinary research team received three hours of training on the study protocol, how to apply inclusion and exclusion criteria correctly, and how to utilize Covidence for scoping reviews. Working in pairs, the full research team independently reviewed and evaluated all retrieved titles/abstracts and then the full text of each article using the aforementioned criteria. Discrepancies during title and abstract screening and full-text review were resolved by the first and second author (T.T., S.S.). Data were extracted from full-text articles using a set of 20 predefined fields related to the study design, methods, outcomes, and implications; intervention characteristics, components, and approach; and study sample characteristics, size, and retention rates. Members of the research team independently extracted data from each article. The first, second, and third authors (T.T., S.S., C.M.) reviewed all extracted data for accuracy and completeness. Consistent with the scoping review methodology [34,40] we did not conduct a formal quality assessment or exclude studies based on methodological rigor. However, we extracted data on the methods used to describe the study design and scope of the intervention. We employed a descriptive approach to synthesize the extracted data, aiming to address the three research questions.

## 3. Results

### 3.1. Study Selection and Characteristics

**Figure 1.**
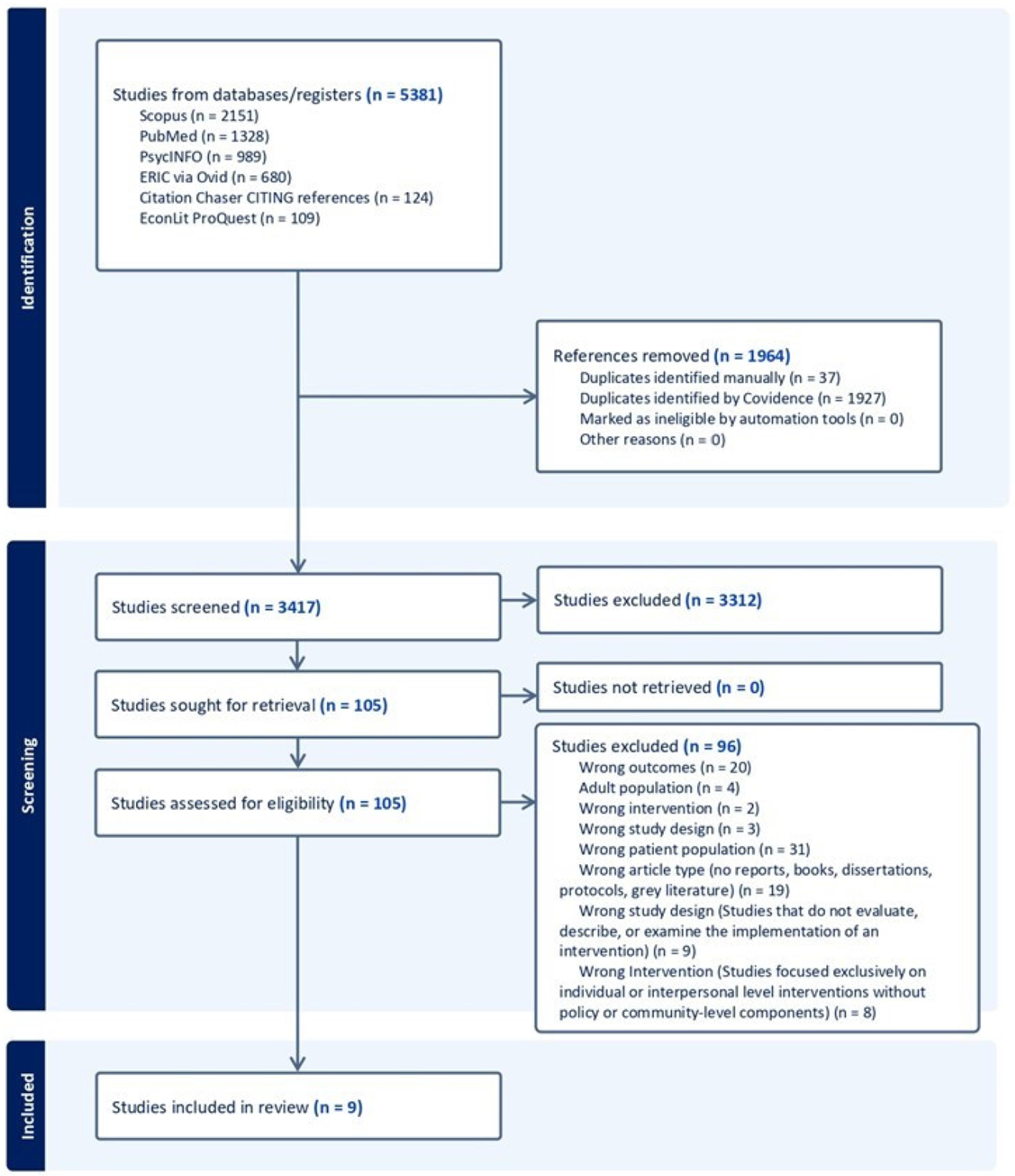
PRISMA diagram summarizing results from the search and screening.

The PRISMA diagram, presented in Figure 1, summarizes results from the search and screening. In total, after removing duplicates, 3417 abstracts were screened for relevance. Of these, 105 full texts were assessed, and nine studies met the inclusion criteria for this review (for details, see the Appendix A). Most studies were excluded because they did not measure health outcomes, did not include the target population, or focused exclusively on individual or interpersonal level interventions.

Table 1 provides an overview of the nine included studies. Most studies were published recently, between 2010 and 2025, and were geographically concentrated in urban areas within the Midwest and Southern US, including Tennessee, Georgia, Minnesota, and Chicago. With the exception of Abraczinskas & Zarrett (2020), most participants lived in low-income areas [41].

Interventions were categorized by mechanism, with seven studies focusing on neighborhoods and social integration, one school-based intervention, and one resource-based intervention. Among the neighborhood and social integration intervention studies, six were interventions that sought to provide health information, social connection building, life skills development, civic engagement, and/or social justice within communities [41–46]. The seventh study implemented policies and environmental interventions that affect the availability of green spaces, walkability, and physical activity [47]. The resource-based intervention provided housing vouchers to Section 8, (a federal assistance housing program) recipients [48], while the school-based study focused on increasing cultural humility and reducing implicit bias and racial microaggressions among teachers serving minority students [49].

### 3.2. Study Samples and Demographics

All nine studies reported participant characteristics. Sample sizes varied substantially, ranging from 21 [42] to 9701 participants [43]. Three studies included only Black participants [42,44,46] while the remaining six studies engaged samples that were predominantly Black (exceeding 60% of the sample). Sex composition varied across studies. Two studies focused exclusively on males [43,46]. Among mixed-sex studies, Kogan et al. (2023) [44] and Krasnova et al. (2025) [48] included nearly equal male and female participants. Abraczinskas and Zarrett (2020) [41] reported a higher proportion of female participants, and Heath and Bilderback (2019) [47] had a higher representation of male participants (approximately 58–59%). None of the included studies reported the sexual orientation of participants. Four studies reported age demographics with mean ages of 11–12 years [41,42,44,46].

### 3.3. Study Purposes and Designs

All nine studies evaluated intervention effects [41–49], with two explicitly assessing intervention feasibility [41,42]. Study designs varied, with three studies using randomized assignment to the intervention or a control group [44,48,49]. Among these were a randomized clinical trial [44] a randomized housing experiment [48] and a waitlist-controlled trial [49]. Five studies used pre-post intervention designs without control groups [42,43,45–47].

### 3.4. Intervention Settings and Community Engagement

Interventions were conducted in various settings including religious settings (n = 2) [42,46], schools (n = 1) [49], community-based organizations (n = 1) [44], clinical community settings (n = 1) [43], general community settings (n = 1) [48] and combined community and school settings (n = 2) [41,45]. Most of the studies included some form of community engagement, although the depth and structure of this engagement varied. In two studies, a community advisory board was established prior to the development of the intervention, with community members providing input throughout the design and implementation process [42,49]. In two other studies, community members were recruited to serve as trainers and outreach workers in their communities [43,44]. Two additional studies engaged student participants in community-based projects as part of a structured educational program [45,46]. Intervention implementation also varied in terms of duration and intensity.

### 3.5. Intervention Components and Durations

Six studies involved educational programs with structured or semi-structured curricula [41,42,44–46,49]. Of these, one study focused on cultural humility training for school staff and teachers, rather than intervention components targeting adolescents directly [49]. The remaining five targeted youth directly.

Two studies were conducted in both school and community environments [41,45]. Connect through PLAY engaged student participants in weekly photovoice and youth-participatory action research activities in order to increase physical activity and green spaces in their communities [41]. The ACHIEVE program paired high school students with undergraduate mentors for a semester-long partnership addressing community health and leadership [45].

Two studies were implemented in religious settings, focusing on skill building and emotion regulation [42], and building life skills for overall health and well-being [46]. The Strong African American Families (SAAF) program was implemented in a community-based organization addressing parenting, self-regulation, and Black pride [44].

All six educational program interventions included in-person components, and three of these interventions also included a virtual or technology-based component [44,45,49].

The remaining three studies employed non- or mixed-educational approaches, including providing housing vouchers [48], STI screening through community outreach [43] and policies and environmental interventions that affect green space, walkability, and physical activity [47]. Intervention duration varied, with some studies describing weekly components (n = 3) and others spanning months to three years (n = 6).

### 3.6. Theoretical Frameworks

Interventions varied in the extent to which they articulated explicit use of relevant theory, although most grounded their approaches in culture, context, or systems-centered theoretical models. The SAAF program was developed using a family-centered preventive intervention model informed by research on parenting, racial socialization, and stress regulation, from a family stress and resilience perspective designed to buffer the effects of racial discrimination on adolescent well-being [44]. Youth-focused interventions frequently use empowerment and justice-oriented theories. Lift Every Voice drew explicitly on Social Justice Service Learning, Positive Youth Development, and youth–adult partnerships to strengthen civic awareness and social responsibility [42]. Similarly, the ACHIEVE program relied on youth–adult partnerships and youth civic engagement frameworks, positioning adolescents as co-creators in addressing community disparities [45]. One study combined Self-Determination Theory with Youth Participatory Action Research and Freirean critical pedagogy to promote autonomy, competence, relatedness, and sociopolitical development [41].

Several interventions used community-level or structural approaches rather than theories based in the fields of health behavior or psychology. One study applied a built environment change framework using systematic observation methods to assess how policy and environmental changes influence youth physical activity [47]. Another study grounded its approach in the socioecological model and a culturally responsive, trauma-informed care framework, implemented through community-engaged research methods to address racial inequities in school climate [49]. Housing-voucher research drew on neighborhood effects and social-causation frameworks [48]. Additional studies relied on culturally grounded or systems-change models, including an African-centered rites of passage model within Positive Youth Development [46] and a harm-reduction, community–university partnership model for STI outreach [43]. While the explicit use of theory varied, many of the included studies relied heavily on evidence from cultural, ecological, and civic frameworks designed to address systems-level inequities. The theoretical foundations of these studies were diverse, which was also reflected in the different types of health outcomes they assessed.

### 3.7. Study Measures and Health Outcomes

All nine studies measured health outcomes, though the types of outcomes varied. Three interventions assessed psychosocial outcomes through youth capacity for community leadership by developing a sense of community attachment and an understanding of systemic inequality, using validated scales including the Psychological Sense of School Membership scale, Social Responsibility scale, and Youth Empowerment scale [41,42,45] Three studies focused on racism-related outcomes, examining perceived racial discrimination or racial bias [46,48,49]. These outcomes are directly related to adolescent health, given the well-established evidence showing the association between exposure to discrimination and health. Three studies examined direct health outcomes, including depressive symptoms [44]. STI screening [43] and observed physical activity levels [47].

Regarding retention, the lowest reported intervention completion rate was 42% [45] while one study reported nearly 92% retention [44]. Six studies reported 100% retention, though this statistic should be interpreted cautiously given the varied intervention designs [41,43,46–49]. More specifically, there were five studies to which attrition was not applicable. One study involved systematic observation of public spaces; therefore, there was no participation attrition [47]. Another study utilized data from the Moving to Opportunity initiative, and although a maximum of 68% of participants used the intervention, all participants were included in the analysis [48]. The intervention implemented by Johnson et al. (2010) [43] utilized street-based outreach rather than formal study enrollment.

### 3.8. Key Findings and Impact

In general, the interventions included in this review had an impact on disrupting SRD and improving Black adolescent health outcomes. Community-based programs, including those with components to engage families, buffered the negative effects of discrimination on mental health, with two studies demonstrating how culturally grounded interventions eliminated or reduced the associations between racial discrimination and depressive symptoms or low self-esteem [44,46]. Next, interventions that centered youth empowerment through the application of participatory and social justice-oriented frameworks (n = 3) showed improved psychosocial outcomes by empowering adolescents to see themselves as change agents, strengthening critical consciousness and community connection while addressing mental health [41,42,45]. These studies also showed that youth-generated, systems-level solutions can address inequities typically overlooked by adults [41]. Finally, interventions that improved the built environment, housing, school-based bias reduction, and community health outreach demonstrated how these methods can remove structural barriers and expand access to health-promoting resources [43,47–49].

## 4. Discussion

In this scoping review, we systematically synthesize the evidence on policy and community-level interventions designed to disrupt SRD exposure among Black adolescents in the US and mitigate its impact on their health behaviors and outcomes. This scoping review identified nine studies examining systems-level interventions: one resource-based intervention [48], seven neighborhood and social integration interventions [41–47]; and one school-based intervention [49]. Despite the small number of studies identified in this review, these studies highlight several effective strategies that disrupt SRD and improve Black adolescent health outcomes. Together, these findings underscore the potential of culturally grounded, multilevel interventions to reduce inequities across mental health, physical health, and social outcomes for marginalized youth and families. These findings also highlight the need to expand systems-level interventions that address the root causes of the persistent racial health inequities experienced by Black youth.

Three patterns emerged from this review that serve as key findings. First, although the review was developed to identify three potential intervention approaches, the majority of the identified studies focused on neighborhood and social integration interventions. Although important, the limited number of resource-based and school-based interventions is a research gap. Despite extensive evidence that housing instability, economic insecurity, and educational barriers perpetuate health inequities among Black adolescents [12,17,50], few empirical studies examine systems-level interventions targeting these mechanisms. Interventions, including guaranteed basic income, cash transfers, tuition assistance programs, and increased access to public transportation, remain under-examined, highlighting a gap in research that addresses the economic and educational conditions shaping adolescent health trajectories. Similarly, this review did not identify studies examining enforcement-based policies that directly target discriminatory practices in labor markets, housing, and policing and their effects on adolescent health. While these studies are distinct intervention mechanisms from resource-based approaches, they may complement these efforts by addressing SRD at its source.

Second, school-based interventions are underrepresented in this literature despite schools being central to adolescent development and where adolescents spend most of their time. Given recent calls from public health for schools to serve as health-promotive settings, this gap is notable [51]. For example, exclusionary discipline policies function as primary mechanisms through which SRD operates in schools, disproportionately affecting Black students and contributing to their poor academic and health outcomes [25–30]. Schools that implement restorative justice and discipline reform practices show reductions in suspensions and arrests, particularly for Black students, and improve perceptions of school safety, climate, and belonging [52]. However, the rigorous implementation and evaluation of these approaches with Black adolescents in the US remains limited.

Third, this review shows that the geographic concentration of studies in urban, southern, and Midwest regions in the US limits the generalizability of findings to rural contexts and other regions. SRD manifests differently across different geographic contexts, which are shaped by different historical contexts, policies, social norms, and economic structures [53]. Black youth in rural settings may face distinct manifestations of SRD, including limited access to services, greater social isolation, and challenging community dynamics, yet research in these settings is limited. Thus, there is a need to understand how interventions function in different contexts and to develop strategies for tailoring proven effective approaches to local conditions [54].

Despite promising results from the studies included in this review, methodological and theoretical limitations may constrain efforts to scale up or further develop these interventions. Few studies explicitly grounded their intervention design or evaluation in frameworks that directly address SRD as a fundamental cause of health inequities, such as Critical Race Theory, Fundamental Cause Theory, or other socioecological models that explicitly include racism. This makes it difficult for researchers and practitioners to identify the mechanisms linking intervention components to the structural drivers of health inequities. Future work should prioritize theory-driven intervention development and evaluation that centers the lived experiences of Black adolescents and accounts for the multilevel, multidimensional nature of SRD. Future research can also examine how system-level interventions may vary across demographic compositions, as our study focused solely on Black adolescents.

Methodological factors also limit the evidence base. Only three studies employed randomized designs; sample sizes were often small, and follow-up periods were short. More rigorous intervention evaluation designs, including control groups and random assignment, should be employed. Additionally, large-scale studies are needed to replicate the findings of current research, which often have variable sample sizes. Larger samples are needed to have sufficient power to detect both intervention effects and to isolate mediators and moderators of the interventions, thereby replicating the findings of the current studies. These limitations reflect broader challenges in conducting systems-level intervention research, where the complexity of interventions, the time required to observe meaningful change, and ethical considerations around randomization pose substantial barriers [55,56]. Innovative methodological approaches may help address these challenges. Agent-based modeling and other simulation approaches offer promising alternatives for evaluating complex systems-level interventions by allowing researchers to model heterogeneous populations, test various intervention scenarios, explore long-term effects, and identify unintended effects that are difficult to assess in traditional study designs [57]. Similarly, machine learning approaches can also be used to identify patterns in existing data, which can then inform the targeting and adaptation of interventions [58]. This type of research may also provide insights into long-term impacts, sustainability, and scalability, which would facilitate the translation of findings into policy and practice.

This review offers several strengths, including a comprehensive search strategy across five databases with forward citation chaining and rigorous dual-review screening at all stages. The multidisciplinary research team brought expertise in psychology, sociology, public health, health policy, systems modeling, adolescent health and development, community-engaged research, school-based health, and SRD, which enabled a more nuanced approach to this review and interpretation of findings. The explicit focus on systems-level interventions fills a gap in the literature and distinguishes this work from prior scoping reviews. Despite these strengths, there are a few limitations that warrant consideration. Consistent with the scoping review methodology, we did not conduct a quality assessment or exclude any studies based on study design or methodological rigor. While this approach is comprehensive, it does mean we are limited in the conclusions we can draw based on the strength of the evidence for these interventions. Additionally, scoping reviews do not include meta-analysis or an assessment of intervention effectiveness, limiting conclusions about which specific intervention components are most impactful in disrupting SRD. The literature search was limited to English-language peer-reviewed publications, potentially excluding relevant grey literature, policy briefs, and community-based evaluations of systems-level interventions.

## 5. Conclusions

The findings from this review underscore the need to expand rigorous research on systems-level interventions to address SRD and improve the health of Black adolescents. Findings suggest several next steps including: (1) development and evaluation of resource-based interventions including guaranteed income, housing assistance, and expanded educational opportunities; (2) implementation research on school discipline reform and other restorative justice practices with attention to racial equity and academic achievement; (3) more efforts to expand research to rural and other understudied geographic contexts; and (4) greater integration of relevant theoretical frameworks and innovative methodological approaches that may facilitate translation of this work. While the small number of studies and sample sizes in this scoping review make it difficult to make strong conclusions, the urgency of the persistent health inequities that Black youth face demands action. Actions should be based on emerging evidence, while also building the knowledge base through rigorous evaluation. Community organizations implementing promising programs need resources, school districts reforming discipline policies need guidance, and policymakers proposing structural reforms need actionable evidence. Dismantling SRD and its health consequences for Black adolescents will require sustained, multisectoral efforts at the policy and community levels that are resilient to shifting social or political priorities.

## Supplementary Material

supplementary material

The following supporting information can be downloaded at: https://www.mdpi.com/article/10.3390/soc16040112/s1, Supplementary Material: 2025-10-23 PubMed search history for Covidence and translations.

## Figures and Tables

**Table 1.** Inclusion and exclusion criteria.

Inclusion Criteria	Exclusion Criteria
Intervention studies that documented the implementation and/or impact of policy and community-level interventions that address or may affect SRD and measure effects on a health outcome	Studies that do not evaluate, describe, or examine the implementation of an intervention
Conducted in the United States	Studies conducted outside of the U.S
Primarily focused on policy and community-level intervention approaches, including resource-based interventions, neighborhood and social integration interventions, or school-based interventions	Studies not written in English
Measured outcomes relevant to adolescents (ages 10–19 years), regardless of whether adolescents are the direct intervention recipients, with a focus on Black populations (i.e., African American, Caribbean Black, African), where at least 51% of the sample is Black	Commentaries, letters to the editor, opinion pieces, dissertations, study protocols, other systematic or scoping reviews, and feature articles (i.e., narrative-style journalistic pieces)
Published in a peer-reviewed journal	Studies focused exclusively on individual or interpersonal level interventions without policy or community-level components.

## Data Availability

No new data were created or analyzed in this study. The original contributions presented in this study are included in the article/Supplementary Material. Further inquiries can be directed to the corresponding author.

## Appendix A

**Table A1.** A summary of the nine studies included in the scoping review.

First Author, Year	Study Purpose	Study Design	Sample Size	Geographic Location	Policy/Intervention Category	Measures Related to Racism and Discrimination	Health Outcomes
Kogan et al., 2023 [44]	“To investigate whether participation in the Strong African American Families (SAAF) program moderates Black adolescents’ depressive symptoms associated with experience of racial discrimination”	Community-based randomized clinical trial; multiple rounds of follow-up	472	7 rural counties in Georgia	Neighborhoods and Social Integration	13-item measure of racial discrimination experiences in the past 6 months. Responses ranged from 1 “never” to 4 “recently”	20-item Scale for Depression Treatment significantly moderated the association of racial discrimination with depressive symptoms, with the treatment group having no significant association between racial discrimination and depressive symptoms
Dinizulu et al., 2024 [42]	To assess the “feasibility and acceptability of a social justice infused service-learning (S-L) program to promote Black adolescent mental health and educational equity”	Open-trial mixed-method design; began by convening a Community Advisory Board (CAB); surveys distributed before, after, and at follow-up; data also collected in follow-up focus-groups with youth; comparison baseline to post-intervention (no control)	21	Mid-western metropolitan neighborhood	Neighborhoods and Social Integration	Post-intervention focus groups explored youths’ awareness of societal and racial inequalities	Significantly positive effects on emotional symptoms, peer problems, and prosocial behaviors, but not for youth self-reports of conduct problems, academic motivation, community belongingness, or social responsibility
Abraczinskas & Zarrett, 2020 [41]	“To address gaps in the youth participation and adolescent physical activity (PA) promotion literature”; and to examine the feasibility of youth participatory action research (YPAR in (a) general aftercare (YPAR only) and (b) with a physical activity intervention, (YPAR + PA) to reach marginalized youth”	Mixed-method concurrent triangulation design. Recruited existing aftercare programs. Two groups: Youth Participatory Action Research (YPAR) with and without Physical Activity (PA); both comparison to baseline and comparison of with/without PA element (no true control)	64	Urban areas	Neighborhoods and Social Integration	Sociopolitical skills reported as youth capacity to identify systemic influences on their health, and barriers and promotors of physical activity in their life, including the influence of power differentials in health decision-making.	Treatment promoted youth empowerment. Youths’ participatory behavior, sociopolitical skills, and perceived control increased
Heath & Bilderback, 2019 [47]	“To assess the impact of physical activity policy and environmental interventions on the physical activity among predominately African American children living in the inner city”	Comparison baseline to post-intervention (no control) system for observing physical activity and recreation in communities observed physical activity by children in public spaces in the community of the intervention	All: year 2010/2011: 838 year 2014: 1091 youth (13–18 y): year 2010/2011: 380 year 2014: 323	Chattanooga, Hamilton County, TN	Neighborhoods and Social Integration	N/A	Authors found support for the idea that improved access to “urban” pedestrian/bicycle routes/trails translate to increased opportunities for physical activity among inner city children/youth
Krasnova et al., 2025 [48]	“To estimate the extent to which [among], non-Hispanic Black and Hispanic adolescents, Section 8 voucher receipt affected the risk of perceived racial/ethnic discrimination in four settings—school, neighborhood, shop/restaurant, or by police—and whether there were differences by sex, city, and voucher type.”	Randomized housing experiment 3 groups; (1) low-poverty voucher, (2) traditional voucher, (3) control all groups measured at baseline and follow-up (4 to 7 years)	2200	Baltimore, Boston, Chicago, Los Angeles, and New York.	Resource-based	1-item measure of adolescents perceived racial discrimination for each setting (school, neighborhood, shop/restaurant, and police)	On average across cities, low-poverty voucher receipt reduced risk of perceived police discrimination among boys and girls. Heterogeneous effects on perceived racial discrimination in various settings across cities. For example, voucher receipt reduced perceived school and neighborhood discrimination in LA but not other cities.
Johnson et al., 2010 [43]	“To examine the effectiveness of providing community-based outreach and health education on increasing sexually transmitted disease (STD) screening”	Comparison to year prior; outreach workers recruited from community	9701	Minnesota	Neighborhoods and Social Integration	N/A	Compared to the year prior to the intervention, actual STD testing of the target population doubled.
Okwumabua et al., 2014 [46]	“To describe how a specific rites of passage intervention, referred to as “the Let the Circle be Un-broken” model (Okwumabua, 1996) [46], is being used to promote the health and well-being of African American children, especially males”	Comparison baseline to post-intervention; surveys administered before program and after program (self-reported) no control group	39	Urban City situated in the Southern US	Neighborhoods and Social Integration	Racial attitudes (pre and post intervention)—the extent to which respondents’ view members of their race positively or negatively	Significant increase on physical self-esteem, a subscale of global self-esteem. Though there was a significant decrease in overall global self-esteem measure. No other significant effects.
Kowalczyk et al., 2024 [45]	“To evaluate the Advancing Community Health and Individual Leadership through a novel educational (ACHIEVE) program”	Pre and post-program surveys, semi-structured interviews (descriptive and thematic analysis), community-based projects. 4 cohorts (but not compared to each other)	85	Chicago’s south side	Neighborhoods and Social Integration	N/A	Nearly all participants reported greater knowledge on health and education issues. Participants also stated they were more knowledgeable about collecting information about health issues in communities and working with others to improve the health of communities.
Ramirez et al., 2023 [49]	“To describe the Link Equity program, its trial design, and the study participants’ baseline characteristics; to reduce the impact of adverse childhood experiences among Black Indigenous and other children of color (BIPOC); and to evaluate Link for Equity’s effectiveness in reducing school violence among BIPOC students”	“Community-engaged waitlist randomized controlled trial,” “nested waitlist-controlled trial,” across 3 pairs of school districts, randomized for intervention or delayed intervention; Community Advisory Board (CAB) began before study, informed study design comparing matched schools (those that received intervention vs. those that were waitlisted) and students	188	Minnesota	School-based	Acceptability of Racial Microaggressions Scale (ARMS) Score from teachers	At baseline, attitudes towards racial microaggressions and trauma-informed care, were very similar between staff at intervention and control schools. American Indian/Alaska Native students generally displayed greater PTSD symptoms than other students
